# Taxing tobacco for better health and higher public revenues: A simulation-based analysis from Serbia

**DOI:** 10.18332/tpc/221526

**Published:** 2026-07-13

**Authors:** Olivera Jovanović, Jovan Zubović, Jovana Strizović

**Affiliations:** 1 Institute of Economic SciencesBelgradeSerbia

**Keywords:** tobacco taxation, cigarette consumption, government revenue, smoking-attributable mortality, public health

## Abstract

**Introduction:**

Tobacco taxation is widely recognized as one of the most effective policy measures for reducing tobacco use and saving lives. The aim of this study is to estimate the fiscal and public health effects of increasing cigarette excise taxes in Serbia, an upper middle-income country with historically high smoking prevalence and increasing affordability in recent years.

**Methods:**

A simulation-based analysis was conducted to estimate the effects of an annual increase in the specific excise tax per cigarette pack in Serbia. The baseline year was 2023, with projections for two years. Changes in cigarette consumption were estimated using price elasticity, income elasticity, and cross-price elasticity derived from previous research. The model assessed impacts on cigarette consumption, government revenues, smoking prevalence, smoking-attributable premature mortality, and youth smoking initiation, under assumptions of full price pass-through, a constant illicit market share, and no inflation.

**Results:**

An increase in cigarette excise taxes is associated with higher retail prices (10–11%) and reductions in cigarette consumption (-4.7% in the first year and -5.3% in the second year) and smoking prevalence (from 37.9% at baseline to 35.8%). Youth smoking initiation declined across all age groups, with >17000 individuals deterred from initiating smoking. Government revenues from tobacco increased over the observed period, as higher tax revenues per pack outweighed reductions in sales volumes, with excise tax revenues rising by about 7% annually. Declines in smoking prevalence resulted, among other effects, in reductions in premature smoking-attributable mortality, with 320 premature deaths avoided in the first year and 678 in the second year.

**Conclusions:**

The findings indicate that increasing cigarette excise taxes in Serbia can yield substantial public health benefits while strengthening government revenues. Further research accounting for substitution toward other tobacco or nicotine products would strengthen the evidence base and provide a more comprehensive assessment of the health and fiscal impacts of tobacco tax policies.

## Introduction

According to the WHO global report on trends in prevalence of tobacco use, the age-standardized prevalence of smoking among individuals aged ≥15 years in Serbia is estimated at 39.7% (39.2% among men and 40.2% among women)^1^. In addition, almost 48% of adults are exposed to tobacco smoke at home, while more than 80% of households with children allow smoking inside the house^2^. Moreover, smoking intensity is also at a high level in Serbia, with about 15.8% of the population aged ≥15 years smoking ≥20 cigarettes per day, compared to only 5.9 in the EU^3^. Despite these indicators, although Serbia performs better than the average of the countries in its income group, it is still one of the countries with the weakest tobacco control policy implementation, relatively low prices and tax share, as well as slow changes in affordability^4^.

Smoking remains a significant public health problem that exacerbates disparities among different income groups. Therefore, a combination of price and non-price tobacco control measures is required to reduce tobacco use^5^. Non-price measures, such as advertising and promotion bans, smoke-free laws, prominent graphic warnings, consumer education campaigns, and smoking cessation therapies, have been shown to reduce the health and economic burden of tobacco use^6^. However, among all tobacco control instruments, excise taxes are considered the most effective in reducing tobacco use and additionally represent an important source of revenue for the government^7^.

In Serbia, tobacco excise policy is governed by the Excise Tax Law, with the Ministry of Finance responsible for its formulation and implementation^4^. Smoking is restricted in workplaces and public premises under the Law on Protection of the Population from Exposure to Tobacco Smoke. Nevertheless, repeated attempts to introduce a comprehensive ban on smoking in cafes, bars, restaurants, and other indoor spaces have not been successful^4^. Recent evidence suggests growing public support for stronger measures, as 40.4% of smokers favor a ban on indoor smoking and vaping⁷, and 40.3% support a 20% price increase if additional revenues are allocated to health, education, or social welfare^8^. Furthermore, the Advertising Law seeks to limit exposure to tobacco promotion, particularly among young people, by imposing strict restrictions on tobacco advertising^4^.

Using fiscal policy as a tool for improving health, the aim of this research is to examine the effects of increases in tobacco excise taxes in Serbia on tobacco consumption, government revenues, and key public health outcomes.

## Methods

### Study design and data sources

**Figure 1:**
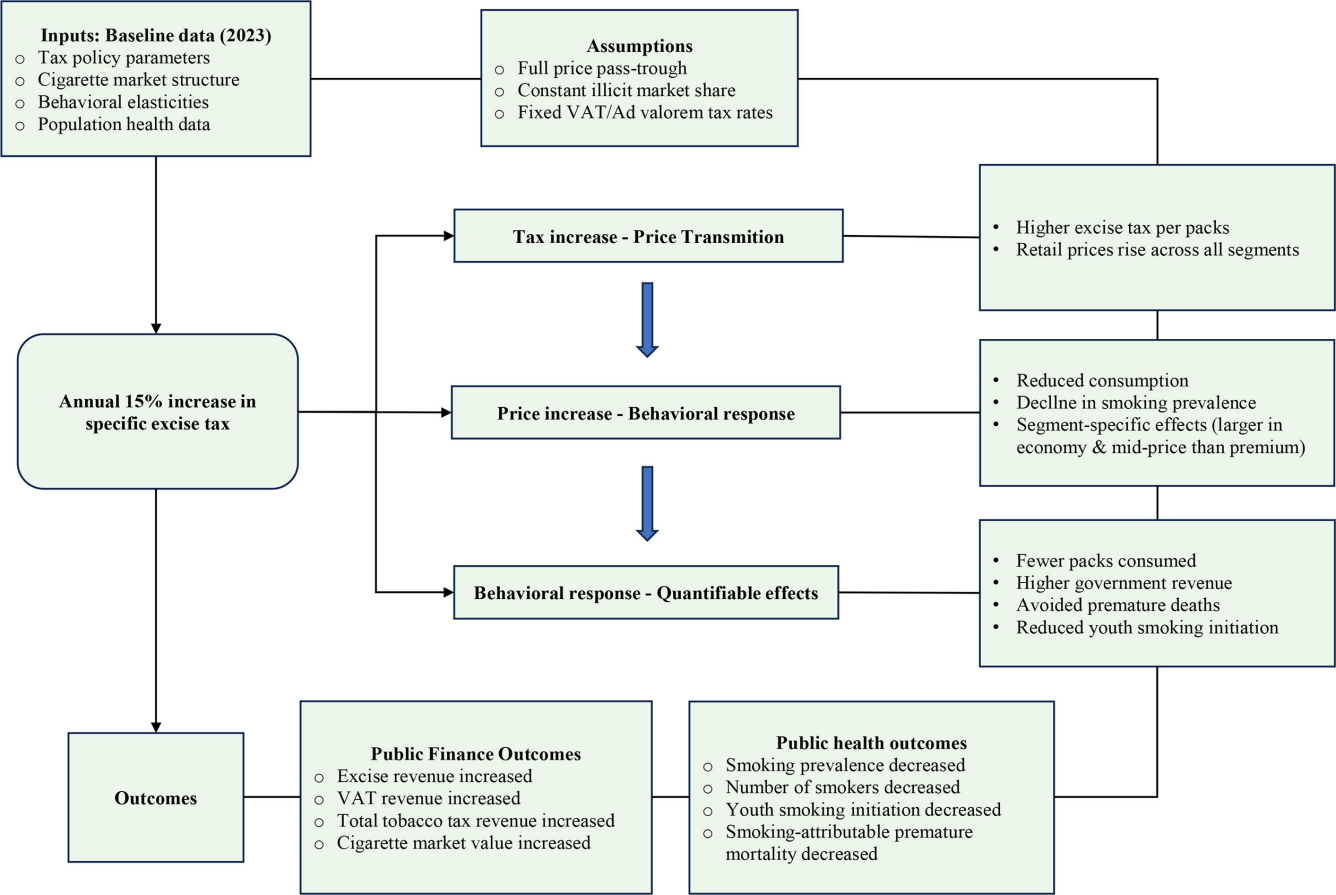
Conceptual framework of the simulation model in Serbia linking cigarette excise tax increases to fiscal and public health outcomes

This study employs a simulation-based analysis to assess the effects of cigarette excise tax increases on tobacco consumption, government revenues, and public health outcomes in Serbia. The analysis focuses on changes in retail cigarette prices resulting from increases in the specific excise tax. The baseline year is 2023, with outcomes simulated for a period of two years. The structure of the simulation model linking excise tax increases to fiscal and public health outcomes is illustrated in Figure 1. All calculations and simulations in this research were performed using Microsoft Excel.

The figure provides a schematic overview of the simulation model used in the analysis. Retail cigarette prices are calculated as the sum of the specific excise tax, ad valorem excise tax, value-added tax (VAT), and the net of-tax component. The model assumes full pass-through of excise tax increases to retail prices, constant ad valorem excise and VAT rates, and a stable illicit cigarette market share over the projection period. Fiscal outcomes include changes in excise tax revenues, VAT revenues, and total tobacco tax revenues, while public health outcomes include changes in smoking prevalence, smoking initiation, and smoking-attributable premature mortality.

The simulation model integrates data from multiple sources. Data on cigarette prices, sales volumes, excise tax rates, and value-added tax were obtained from the Serbian Tobacco Administration and the Ministry of Finance of the Republic of Serbia. Data on cigarette market structure and price segmentation were sourced from Euromonitor. Adult smoking prevalence data were obtained from the Survey on Tobacco Consumption in Southeastern Europe (STC-SEE)^2^, while smoking prevalence among young people was derived from the Serbian National Health Survey^9^ and the Global Youth Tobacco Survey (GYTS)^10^. Population data were provided by the Statistical Office of the Republic of Serbia (SORS), with demographic projections adjusted using International Monetary Fund estimates. Price, income, and cross-price elasticity parameters applied in the model were drawn from previously published studies for Serbia and comparable countries^11,12^. The key baseline inputs and assumptions used in the simulation model are summarized in Table 1.

**Table 1:** Key baseline inputs and assumptions used in the simulation model, Serbia, 2023

Indicator	Value	Source
Specific excise tax	82.75 RSD per pack	Ministry of Finance
Ad valorem excise tax rate	33% of retail price	Ministry of Finance
Value-added tax rate	20% of retail price	Ministry of Finance
Illicit cigarette market share	2.6% of total market	Đukić et al.^16^
Adult smoking prevalence (≥18 years)	37.9%	STC-SEE (2019)
Adult population (≥18 years)	5.63 million persons	SORS, IMF projections
Adult prevalence elasticity (price)	-0.265 (0.05)	Zubović et al.^18^
Price elasticity of demand	Segment-specific values	Zubović et al.^18^
Income elasticity of demand	Segment-specific values	Zubović et al.^18^
Cross-price elasticities	Segment-specific values	Tauras et al.^12^
Youth price elasticity (≤17 years)	-1.44	IARC (2011)
Youth price elasticity (18–24 years)	-0.89	IARC (2011)

RSD: Serbian Dinars, average 2023 exchange rate 1 € = 117.25 RSD, as reported by the National Bank of Serbia. All parameters refer to the baseline year 2023 unless stated otherwise. Segment-specific price, income, and cross-price elasticities used in the simulation model are reported in Supplementary file Table S1.

IARC: International Agency for Research on Cancer; IMF: International Monetary Fund; SORS: Statistical Office of the Republic of Serbia; STC-SEE: Survey on Tobacco Product Consumption in Southeastern Europe.

To better predict the effects of cigarette price changes while taking into account the specific characteristics of smokers’ behavior and consumption patterns, the cigarette market was segmented into three price categories: economy, mid-price, and premium. Due to the absence of official price data by market segment, average retail prices were approximated using cigarette price data by individual brands reported by the Tobacco Administration. The mid-price segment was defined by the most frequently sold brands, while lower priced brands were classified as economy and higher priced brands as premium. All monetary values are expressed in Serbian Dinars (RSD). For the purpose of international comparability, an indicative conversion is provided based on the average 2023 exchange rate of 1 € = 117.25 RSD, as reported by the National Bank of Serbia^13^.

### Simulation of fiscal and public health outcomes

To validate the model linking the effects of increases in the specific excise tax on public health and public finances, we assumed a simulation scenario. In this study, the simulation scenario evaluates the effects of two annual 15% increases in the specific excise tax per cigarette pack, following similar scenario analyses conducted in Western Balkan countries^14,15^ in order to ensure comparability of results and to support policy recommendations aligned with regional trends in tobacco consumption. Retail cigarette prices were calculated as the sum of the specific excise tax, ad valorem excise tax, value-added tax (VAT), and the net-of-tax component. The projections assume constant ad valorem excise and VAT rates throughout the projection period (33% of the retail price and 20%, respectively), along with full pass-through of excise tax increases to retail prices.

Based on available empirical evidence for Serbia, the share of the illicit cigarette market was set at 2.4% of total consumption and assumed to remain constant over the projection period since^16^. Based on existing research evidence, changes in the illicit market share are not driven by increases in tobacco excise taxes but rather by broader structural and institutional factors, including governance quality, enforcement capacity, and the presence of informal distribution channels^17^. Changes in cigarette consumption were estimated using price elasticity, income elasticity, and cross-price elasticity. Because market segment–specific elasticity estimates for Serbia are unavailable, elasticity values by income group from previous national studies were used as proxies for the respective market segments^11^.

Fiscal outcomes were estimated by calculating changes in excise tax revenues, VAT revenues, and total tobacco tax revenues. Excise revenues represent the excise tax per pack multiplied by the number of cigarette packs sold within each market segment, while total tobacco tax revenues were calculated as the sum of excise taxes and VAT across all segments. All fiscal outcomes were estimated in nominal terms. However, as the analysis focuses on proportional changes in revenues following excise tax increases, this does not alter the main conclusions regarding the direction and magnitude of fiscal effects.

Public health impacts were assessed by estimating changes in smoking prevalence, the number of smokers, and smoking-attributable premature mortality. Changes in adult smoking prevalence are based on prevalence elasticities in response to cigarette price changes, while the number of smokers was estimated by applying projected prevalence rates to the adult population. Relative risk and smoking-attributable fraction indicators for Serbia are used to estimate the reduction in premature mortality and are derived from data provided by the Republic Health Insurance Fund of Serbia, based on smoking-related diseases classified according to ICD-10 codes^18^. In the absence of direct empirical evidence on individual mortality outcomes following smoking cessation, the analysis assumes that a fixed proportion of individuals who quit smoking in response to price increases avoid smoking-attributable premature mortality, consistent with previous estimates^14^.

Among public health outcomes, youth smoking initiation is a critical element of tobacco control policy. Nicotine dependence often forms during early adolescence, which emphasizes the need to address this issue through appropriate strategic policy measures in order to achieve the greatest long-term impact on overall wellbeing. Building on previous research, the impact of cigarette price increases on youth smoking initiation was estimated for three age groups: ≤14, 15–17, and 18–24 years. The number of potential new smokers was calculated using age-specific population data and smoking prevalence rates. Reductions in smoking initiation were estimated using youth-specific price elasticities reported in the literature^19^, under the assumption that higher cigarette prices deter or delay the onset of smoking.

## Results

The scenario analysis developed within this simulation model is designed to project two groups of indicators: future trends in key public finance outcomes and public health outcomes. According to available data for Serbia, an annual increase of 15% in the specific excise tax led to higher retail cigarette prices. The baseline cigarette market structure and tax revenue distribution across price segments in 2023 are presented in Table 2. Average cigarette prices increased by approximately 10% in 2024 and by 11% in 2025, followed by a decline in total cigarette consumption. The total number of cigarette packs sold decreased by -4.7% in the first year and by -5.3% in the second year compared with the baseline.

**Table 2:** Baseline cigarette market structure and tobacco tax revenues by price segment, Serbia, 2023

Market segment	Price per pack (RSD)	Packs sold (thousand)	Market share (%)	Total excise revenue (thousand RSD)	Total tax revenue (thousand RSD)
Premium	435.0	141097	23.0	31930344	42159906
Mid-price	325.0	323297	52.7	61426451	78938377
Economy	265.0	149072	24.3	25372136	31956171
Total	-	613467	100	118728931	153054454

RSD: Serbian Dinars, average 2023 exchange rate 1 € = 117.25 RSD, as reported by the National Bank of Serbia. Prices by market segment are approximated using representative brand prices reported by the Tobacco Administration. Market segmentation reflects economy, mid-price, and premium cigarette categories.

Despite the reduction in consumption, government revenues from tobacco increased in both projected years. Detailed fiscal projections for 2024–2025 are shown in Table 3. Total excise tax revenues rose by approximately 7.0% in both 2024 and 2025. In addition, the total market value of cigarette sales increased by 5.1% in 2024 and by 5.4% in 2025 compared with the starting point. Reductions in consumption occurred in all market segments, with relatively larger declines in the economy and mid-price segments, while changes in the premium segment were smaller.

**Table 3:** Estimated public finance effects of cigarette excise tax increases, Serbia, 2024–2025

Indicator	2023 (Baseline)	**2024**	**2025**
Cigarette packs sold (million)	613.47	584.89 (578.28–591.49)	553.81 (541.1–566.7)
Change in packs sold (%)	n/a	−4.7 (3.6–5.7)	−5.3 (4.2–6.4)
Total excise revenue (billion RSD)	118.73	127.06 (125.68–128.43)	135.87 (132.88–138.90)
Change in excise revenue (%)	n/a	7.0 (5.9–8.2)	7.0 (5.7–8.1)
Total tobacco tax revenue (billion RSD)	153.05	163.12 (161.37–164.87)	173.88 (170.09–177.72)
Change in total revenue (%)	n/a	6.6 (5.4–7.7)	6.6 (5.4–7.7)
Total market value (billion RSD)	205.95	216.36 (214.1–218.61)	228.06 (223.23–232.95)
Change in market value (%)	n/a	5.1 (4.0–6.1)	5.4 (4.3–6.6)

RSD: Serbian Dinars, average 2023 exchange rate 1 € = 117.25 RSD, as reported by the National Bank of Serbia. Results are based on a simulated annual 15 percent increase in the specific excise tax, assuming full price pass-through and a constant illicit market share. Percent changes are relative to the baseline year (2023). Value ranges for each indicator were derived from the estimated own-price elasticity ranges for each market group used in calculation.

On the other hand, higher cigarette prices have also generated significant positive effects on public health. An increase in cigarette prices was associated with reductions in adult smoking prevalence in this scenario analysis, declining from 37.9% in the baseline year to 36.9% in the first year and further to 35.8% in the second year, with corresponding reductions in the number of smokers and smoking-attributable premature mortality summarized in Table 4. Consistent with these trends, the total number of adult smokers decreased by approximately -3.1% in the first year and by -3.4% in the second year relative to baseline.

**Table 4:** Estimated public health effects of cigarette excise tax increases, Serbia, 2024–2025

Indicator	**2023** (Baseline)	**2024**	**2025**
Smoking prevalence (%)	37.9	36.9 (36.5–37.3)	35.8 (35.0–36.5)
Number of smokers (million)	2.14	2.07 (2.05–2.09)	2.00 (1.96–2.06)
Reduction in number of smokers (%)	n/a	0.066 (0.045–0.087)	0.07 (0.048–0.092)
Smoking-attributable premature deaths avoided (up-to date)	n/a	320 (224–450)	678 (417–941)
Smoking-attributable premature deaths avoided (lifetime)	n/a	46320 (31461–61179)	49054 (33382–64397)

Estimates of smoking-attributable premature mortality are based on relative risk and smoking-attributable fraction parameters for Serbia. Lifetime mortality reductions assume that 70 percent of individuals who quit smoking avoid smoking-attributable premature death. Value ranges for each indicator were derived from the estimated prevalence elasticity ranges used in calculation.

Reductions in smoking prevalence led to lower smoking-attributable premature mortality. The estimated number of individuals avoiding premature death increased over time, with approximately 320 premature deaths avoided in the first year and 678 avoided in the second year. Lifetime estimates derived from the simulation model indicate a substantial cumulative reduction in smoking-attributable premature mortality among individuals who quit smoking in response to higher cigarette prices.

Cigarette price increases were also associated with reductions in smoking initiation among young people. The estimated number of individuals deterred from smoking initiation or delaying initiation exceeded 17000 across all youth age groups combined. Reductions in smoking initiation were observed across all age categories, with the largest effects among individuals aged 18–24 years, followed by those aged ≤14 years, and those aged 15–17 years.

## Discussion

This study provides simulation-based evidence that increases in cigarette excise taxes in Serbia lead to higher retail prices, reduced cigarette consumption, and lower smoking prevalence, while simultaneously increasing government revenues from tobacco. Specifically, two consecutive annual increases of 15% in the specific excise tax were associated with price increases of approximately 10–11%, declines in cigarette sales of 4.7–5.3%, and reductions in adult smoking prevalence from 37.9% to 35.8%. Despite lower consumption, excise tax revenues increased by around 7% annually, and meaningful public health gains were observed in the form of reduced smoking-attributable premature mortality and lower youth smoking initiation. These findings provide the basis for comparison with existing evidence from Serbia and other countries, discussed below.

The results indicate that higher cigarette excise taxes are associated with an increase in retail prices and reduced cigarette consumption, while government revenues from tobacco continue to rise. This pattern is consistent with evidence from a range of settings, including Southeastern Europe and other low- and middle-income countries^20^. In such contexts, higher tax revenues per pack tend to outweigh reductions in sales volumes, resulting in net revenue gains following excise tax increases.

In policy debates, the tobacco industry frequently argues that higher excise taxes reduce government revenues due to declining consumption and the expansion of illicit trade. Despite reductions in cigarette consumption, tobacco tax revenues increased over the projection period, indicating that the increase in tax revenue per pack more than compensated for declines in sales volumes under conditions of a stable illicit market share. Similar patterns have been documented both in the region and more broadly, with simulation-based and empirical studies from neighboring countries^14,15^, as well as from economies such as Nigeria^21^ and South Africa^22^, showing that excise tax increases were not followed by sustained declines in government revenues^20^.

From a public health perspective, the estimated reductions in smoking prevalence align with extensive evidence showing that higher cigarette prices encourage smoking cessation and reduce smoking intensity^23,24^. Previous research has shown that price responsiveness varies across population groups, with more price-sensitive smokers contributing disproportionately to reductions in prevalence^25^. The magnitude and direction of the reductions observed in this study are consistent with this body of evidence.

The analysis further indicates that cigarette price increases are accompanied by reductions in smoking initiation among young people. International evidence consistently demonstrates that youth are particularly responsive to price changes^26-28^. By discouraging or delaying smoking initiation, tobacco excise taxation contributes not only to immediate reductions in smoking prevalence but also to long-term declines in tobacco-related mortality.

Estimated reductions in smoking-attributable premature mortality highlight the broader health benefits of tobacco taxation. As in other simulation-based studies, assumptions regarding mortality risk following smoking cessation are required due to the absence of individual-level longitudinal data^14^. While this introduces uncertainty, the use of transparent and conservative assumptions allows for meaningful comparisons with existing studies and supports interpretation of the results.

### Strengths and limitations

An important contribution of this study lies in demonstrating the applicability of a simulation-based framework for assessing the combined fiscal and public health effects of tobacco excise taxation. Although the projections refer to 2 years, the objective of the analysis is to examine the internal coherence and policy relevance of a modeling framework that links changes in tobacco excise taxes to cigarette prices, consumption, government revenues, and health outcomes. This approach is particularly relevant in the Serbian context, where high smoking prevalence, a substantial tobacco-related disease burden, and the significant fiscal role of tobacco taxation create a strong need for integrated analytical tools to support evidence-based policy decisions. By providing a transparent and structured representation of key policy mechanisms, the model offers a flexible framework that can be updated as new data become available and extended to alternative tax scenarios. As such, it represents an early-stage analytical tool establishing proof of concept for integrated fiscal - health policy modeling in Serbia and laying the groundwork for more advanced applications in future tobacco tax policy analysis.

Several limitations should be acknowledged. The analysis relies on assumptions regarding price pass-through, illicit trade, and behavioral elasticities, which may evolve over time. Substitution toward other tobacco or nicotine products is not explicitly modeled, and health outcomes are estimated using aggregate parameters rather than individual-level data, which should be considered when interpreting the findings. The simulation model provides scenario-based projections rather than causal estimates, as the results depend on predefined assumptions and do not capture all real-world determinants. Even though the results come from Serbia, this model can also be applied to data from other middle-income countries, provided that country-specific indicators are incorporated at the individual level, while accounting for the mixed system of tobacco taxation.

### Implications

Taken together, the findings suggest that increases in cigarette excise taxes can reduce tobacco use, discourage smoking initiation, and lower smoking-attributable mortality while maintaining or strengthening government revenues. Tobacco excise taxation therefore emerges as a policy instrument capable of aligning fiscal objectives with public health goals in Serbia.

## Conclusions

This study shows that increasing the specific excise tax on cigarettes in Serbia can simultaneously advance fiscal sustainability and public health objectives. The simulation-based analysis indicates that higher excise taxes are associated with reduced cigarette consumption, lower smoking prevalence, and meaningful declines in smoking-attributable premature mortality, while government revenues from tobacco are maintained or increased.

By demonstrating that revenue gains can coexist with substantial health benefits, the findings challenge common arguments against tobacco tax increases and support the role of excise taxation as a core tobacco control measure. The results highlight the value of simulation-based tools for informing evidence-based fiscal and health policy decisions, particularly in settings with high smoking prevalence.

Overall, tobacco excise taxation emerges as a policy instrument capable of protecting population health while strengthening public finances, offering a clear opportunity to align economic and public health priorities in Serbia. Further independent research on modeling the impacts of tobacco taxation is needed, especially to incorporate the modeling of health taxes on other products associated with risky health behaviors. The development of a micro-simulation model would be beneficial for policymakers in Serbia, as it would significantly improve the robustness of estimated effects of excise tax changes not only on tobacco products but also on other related products.

## Abbreviations

EU: European Union
EUR: Euro
GYTS: Global Youth Tobacco Survey
IARC: International Agency for Research on Cancer
ICD-10: International Classification of Diseases, 10th Revision
IMF: International Monetary Fund
RSD: Serbian Dinar
SORS: Statistical Office of the Republic of Serbia
STC-SEE: Survey on Tobacco Consumption in Southeastern Europe
UIC: University of Illinois Chicago
VAT: Value-Added Tax
WHO: World Health Organization

## References

[1] World Health Organization (2025). WHO global report on trends in prevalence of tobacco use 2000–2024 and projections 2025–2030.

[2] Zubović J, Vladisavljević M, Đukić M (2019). Survey on tobacco products consumption in Southeastern Europe (STC-SEE). http://dcs.ien.bg.ac.rs/61.

[3] Vladisavljevic M, Zubović J, Jovanovic O, Đukić M (2024). Crowding-out effect of tobacco consumption in serbia. Tob Control.

[4] Zubović J, Vukmirović V, Jovanović O (2026). Tobacco taxation in Eastern Europe – landscape study. Institute of Economic Sciences.

[5] Hiscock R, Bauld L, Amos A, Fidler JA, Munafò M (2012). Socioeconomic status and smoking: A review. Ann N Y Acad Sci.

[6] World Health Organization (2003). WHO Framework Convention on Tobacco Control.

[7] Chaloupka FJ, Yurekli A, Fong GT (2012). Tobacco taxes as a tobacco control strategy. Tob Control.

[8] Jovanovic O, Zubovic J (2023). Smokers’ attitudes on control policies and an overview of the current state in Serbia. Tob Prev Cessation.

[9] Institute of Public Health of Serbia (2021). Statistical Office of the Republic of Serbia. The 2019 Serbian National Health Survey.

[10] World Health Organization (Updated July 31, 2015). Serbia 2013: Global Youth Tobacco Survey (GYTS) Fact Sheet (Ages 13–15). https://www.who.int/publications/m/item/2013-gyts-fact-sheet-serbia.

[11] Zubović J, Vladisavljević M (2019). Impact of tobacco excise increases on cigarette consumption and government revenues in Southeastern Europe countries. Institute of Economic Sciences.

[12] Tauras JA, Peck RM, Chaloupka FJ (2006). The role of retail prices and promotions in determining cigarette brand market shares. Rev Ind Organ.

[13] National Bank of Serbia (2026). Average annual exchange rate of the dinar (RSD) against the euro (EUR) for 2023.

[14] Borović Z, Mićić L, Gligorić D, Preradović Kulovac D (2023). Bosnia and Herzegovina tobacco excise tax modeling.

[15] Cizmovic M, Mugosa A, Kovacevic M, Lakovic T (2022). Effectiveness of tax policy changes in Montenegro: Smoking behaviour by socio-economic status. Tob Control.

[16] Đukić M, Jovanović O, Vladisavljević M, Jolović N, Zubović J (2021). Tobacco tax avoidance and evasion in Serbia, 2019.

[17] Vladisavljević M, Đukić M, Zubović J, Jovanović O, Jolović N (2021). Tobacco Tax Evasion in Southeastern Europe: Tax Evasion Prevalence and Evasion Determinants.

[18] Zubović J, Jovanović O, Nedeljković B (2024). Distributional impacts of tobacco excise taxes in Serbia. Nicotine Tob Res.

[19] International Agency for Research on Cancer (2011). Volume 14: Effectiveness of Tax and Price Policies for Tobacco Control.

[20] Goodchild M, Perucic AM, Nargis N (2016). Modelling the impact of raising tobacco taxes on public health and finance. Bull World Health Organ.

[21] Bardach A, Casarini A, Rodriguez Cairoli F (2022). The estimated benefits of increasing cigarette prices through taxation on the burden of disease and economic burden of smoking in Nigeria: A modeling study. PLoS One.

[22] Doherty J (2014). Increasing tax revenue and its impact on financing public health care in South Africa. RESYST Working Paper No. 6.

[23] Matsubayashi K, Tabuchi T, Iso H (2021). Tobacco price increase and successful smoking cessation for two or more years in Japan. Nicotine Tob Res.

[24] Ross H, Blecher E, Yan L, Hyland A (2011). Do cigarette prices motivate smokers to quit? Evidence from the United States and Canada. Addiction.

[25] Tsai YW, Yang CL, Chen CS, Liu TC, Chen PF (2005). The effect of Taiwan’s tax-induced increases in cigarette prices on brand-switching and the consumption of cigarettes. Health Econ.

[26] Parks MJ, Patrick ME, Levy DT (2022). Cigarette pack price and its within-person association with smoking initiation, smoking progression, and disparities among young adults. Nicotine Tob Res.

[27] Kostova D, Ross H, Blecher E, Markowitz S (2011). Is youth smoking responsive to cigarette prices? Evidence from low- and middle-income countries. Tob Control.

[28] Filby S, van Walbeek C (2022). Cigarette prices and smoking among youth in 16 African countries: Evidence from the Global Youth Tobacco Survey. Nicotine Tob Res.

