# Supplementary material for: Taxing tobacco for better health and higher public revenues: A simulation-based analysis from Serbia
Source: Tob Prev Cessat. 2026 Jul 13;12:10.18332/tpc/221526. doi: 10.18332/tpc/221526 (PMC13365776; doi:10.18332/tpc/221526)
Supplement: Supplementary file Table S1 [file TPC-12-39-s001.pdf]

TOBACCO PREVENTION AND CESSATION

Supplementary file

© 2026 Jovanović O. et al.

DOI:
10.18332/tpc/221526

The content has been provided by the author(s) and has not been reviewed, verified, or endorsed by European Publishing. It may not have undergone peer review. The views, opinions, and recommendations expressed are solely those of the author(s) and do not necessarily reflect the position of European Publishing. European Publishing accepts no responsibility or liability for any consequences arising from the use of, or reliance on, this content.

Supplementary file Table S1.

Table S1. Price, income, and cross-price elasticities used in simulation-based analysis

Indicator	Market segments		
	Premium	Mid-price	Economy
**Price elasticity**	-0.220	-0.631	-1.076
**Income elasticity**	0.740	1.267	1.363
**Cross-price elasticity premium**		0.150	0.010
**Cross-price elasticity mid-range**	0.620		0.060
**Cross-price elasticity economy**	0.270	0.130	
